# NOD2 Agonist Promotes the Production of Inflammatory Cytokines in VSMC in Synergy with TLR2 and TLR4 Agonists

**DOI:** 10.1100/2012/607157

**Published:** 2012-09-10

**Authors:** Jinghua Sun, Yanchun Ding

**Affiliations:** ^1^Department of Oncology, The Second Affiliated Hospital of Dalian Medical University, Dalian 116027, China; ^2^Department of Cardiology, The Second Affiliated Hospital of Dalian Medical University, Dalian 116027, China

## Abstract

*Objective*. To investigate the expression of NOD2 in human VSMCs, its role in the production of inflammatory cytokines in VSMC and the possible interaction of NOD2-mediated signaling pathway with those mediated by TLR2 and TLR4. *Methods*. Human coronary artery smooth muscle cells were stimulated with NOD2 agonist MDP alone or in combination with either TLR2 agonist PAM3 or TLR4 agonist LPSs. The mRNA expression of NOD2 and FGF-2 were measured by RT-PCR. The concentration of IL-8 and TNF-**α** in the culture supernatants was determined by ELISA. VSMC proliferation ability was analyzed by MTT assay. *Results*. MDP up regulated the expression of NOD2 mRNA in VSMC in a time-dependent manner, up regulated the expression of FGF-2 mRNA in VSMC, induced the production of IL-8 and TNF-**α**, and promoted the proliferation of VSMC. Additionally, MDP synergied with LPS and PAM3 to promote the proliferation of VSMC and induce the production of IL-8 and TNF-**α**. *Conclusion*. The activation of NOD2-mediated innate immune signaling pathway can increase the proliferation ability of VSMC and induce the production of inflammatory cytokines in VSMC. It is also shown a synergistic effect with TLR2- and TLR4-mediated signaling pathways in this process.

## 1. Introduction

Toll-like receptors (TLRs) and nucleotide-binding oligomerization domains (NODs) are two pattern-recognition receptors (PRR) families related to innate immune defense. TLRs located on the cell surface are responsible for sensing extracellular dangerous signals [1], while NODs are cytosolic proteins involved in intracellular recognition of microbes and their products [2]. Both receptors can activate the nuclear factor-*κ*B (NF-*κ*B) pathway to induce the production of inflammatory cytokines and initiate the innate immune response of the host cells to various pathogen infections. Studies have shown that TLRs-mediated innate immune signaling pathways play an important role in cardiac hypertrophy and arterial atherosclerosis [3], and they are involved in the regulation of proliferation, differentiation, and apoptosis of vascular smooth muscle cells (VSMCs). However, the function of NODs in these processes and their relationship with the TLRs signaling pathways is poorly understood. In this paper, we studied the expression of NOD2 and the function of NOD2-mediated innate immune signaling pathway in the production of inflammatory cytokines in VSMC, and we further explored its relationship with TLR2, 4-mediated innate immune signaling pathway in this process.

## 2. Materials and Methods

### 2.1. Cell Lines

Human vascular smooth muscle cells were purchased from Cambrex (Walersville, USA).

### 2.2. Reagents

MDP, PAM3, LPS, and DMEM medium were purchased from InvivoGen (San Diego, USA); fetal bovine serum was purchased from Gibco BR; RNeasy total RNA mimi Kit was purchased from Qiagen Inc. (California, USA); ELISA Kit of IL-8 and TNF-*α* was from BD Phamingen.

### 2.3. Cell Culture 

Human vascular smooth muscle cells (VSMCs) were cultured in DMEM medium containing 10% fetal bovine serum, 100 U/mL penicillin, 100 U/mL streptomycin, and 1 mmol/L L-glutamine in an incubator with 5% carbon dioxide at 37°C. VSMCs on log-growth were further transplanted into DMEM medium containing 0.1% fetal bovine serum, seeded again in 6-well plates on concentration of 1 × 10^6^/well, and cultured for 24 hours. Then, cells were treated with either MDP (10 ng/mL) alone for 0, 3, 6, and 24 hours, or with MDP (10 ng/mL), LPS (1 *μ*g/mL), and PAM3 (10 ng/mL) alone or in combination for 24 hours, while no stimulant was added into as control group.

### 2.4. MTT Assay 

VSMCs were seeded in 96-well plates. 80% confluent VSMCs were synchronized for 24 hours and treated with vehicle-control or different drugs (6 wells for each group). After 24 hours, MTT (5 mg/mL) was added to each well. Cells were incubated for another one hour and then made soluble with cytolysis solution (10% Triton X-100, 0.1 mmol/L HCl in isopropyl alcohol solution). Absorbance was determined at 570 nm by spectrophotometry. 

### 2.5. RT-PCR Analysis

Total RNA was prepared using the RNeasy Mini Kit (Qiagen, Valencia, CA). Purified RNA was reverse transcribed with BioAnalyzer (Agilent Technologies, Germany) according to the manufacturer's protocol. The generated cDNA was amplified by using primers (all from Assay-on-demand, ABI, Foster City, California, USA) for human NOD2, FGF-2, and cyclophinlin A. 

### 2.6. ELISA

The concentration of IL-8 and TNF-*α* in the culture supernatants was determined by ELISA (BD Phamingen) as specified by the manufacturer.

### 2.7. Statistical Analysis

Results are reported as mean ± SEM. Statistical analysis was performed using SPSS 10.0 software, and one-way ANOVA was used within groups. *P* < 0.05 was considered significant.

## 3. Results

### 3.1. MDP-Induced Expression of NOD2 mRNA in VSMC

**Figure 1:**
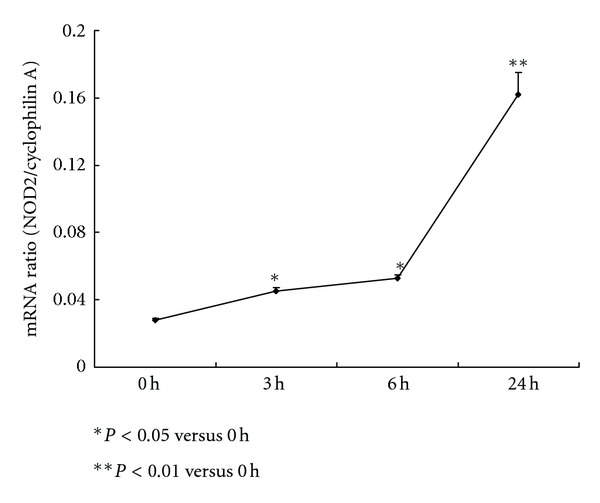
MDP induced the expression of NOD2 in VSMC. MDP stimulation (10 ng/mL) caused time-dependent increase of NOD2 mRNA expression in VSMC and reached a peak at 24 h (0 h: 0.028 ± 0.001, 3 h: 0.045 ± 0.002; 6 h: 0.053 ± 0.002; 24 h: 0.162 ± 0.013; *P < 0.05, **P < 0.01, *n* = 3). NOD2 mRNA relative expression level = NOD2/cyclophilin A.

RT-PCR analysis showed lower expression level of NOD2 in VSMC in basic state. However, after MDP stimulation, the expression level increased in a time-dependent manner and reached maximal value at 24 h (see Figure 1).

### 3.2. MDP Stimulated VSMC to Produce Inflammatory Cytokines

**Figure 2:**
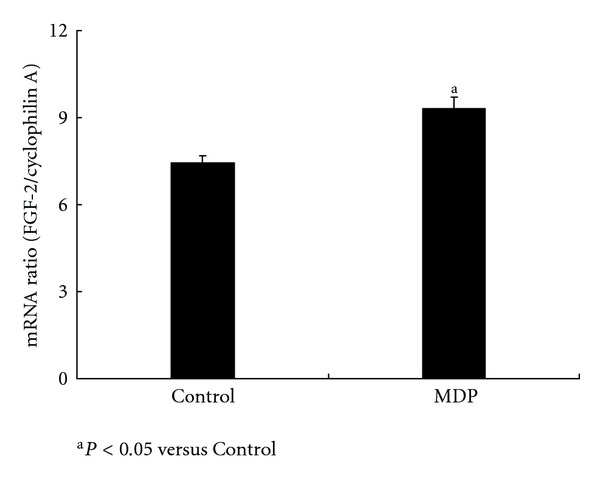
MDP stimulated FGF-2 mRNA expression in VSMC. FGF-2 mRNA expression increased in VSMC after 24-h MDP (10 ng/mL) stimulation (9.32 ± 0.37 versus control 7.44 ± 0.25; ^a^ *P* < 0.05, *n* = 3). FGF-2 mRNA relative expression level = FGF-2/cyclophilin A.

To further explore the function of NOD2 in VSMC, we used MDP to stimulate VSMC for 24 h. After stimulation, FGF-2 mRNA expression was unregulated in VSMC, and in the culture supernatant, the concentration of IL-8 and TNF-*α* mRNA expression increased as well, indicating that NOD2 activation can promote VSMC to secrete inflammatory cytokines (see Figures 2, 4(a), and 4(b)).

### 3.3. Proliferation Ability of VSMC in Response to MDP Alone or in Synergy with LPS and PAM3

**Figure 3:**
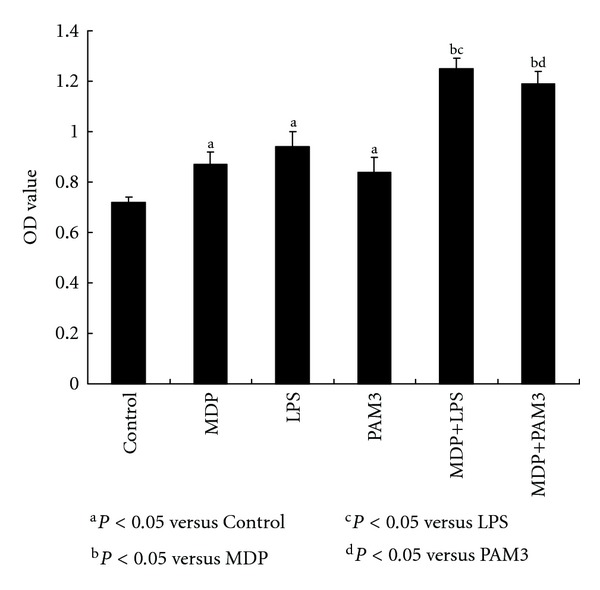
Proliferation ability of VSMC in response to MDP alone or in synergy with LPS and PAM3. MTT assay was done to test the proliferative activity of VSMC. MDP, LPS, or PAM3 alone can stimulate VSMC proliferation ability (MDP: 0.87 ± 0.05; LPS: 0.94 ± 0.06; PAM3: 0.84 ± 0.06; control: 0.72 ± 0.02; ^a^ *P* < 0.05 versus control, *n* = 6). The combination of MDP with LPS or PAM3 got more activated proliferation ability (MDP + LPS: 1.25 ± 0.04; MDP + PAM3: 1.19 ± 0.05; ^b^ *P* < 0.05 versus MDP; ^c^ *P* < 0.05 versus LPS; ^d^ *P* < 0.05 versus PAM3; *n* = 6).

We used the MTT assay to detect proliferation ability of VSMC with MDP, LPS, or PAM3 treatment alone or in combination. All three reagents can promote VSMC proliferation. Treatment by MDP combined with either LPS or PAM3 got higher proliferation ability than MDP, LPS, or PAM3 alone (see Figure 3).

### 3.4. Inflammatory Factors Production by VSMC in Response to MDP Alone or in Synergy with LPS and PAM3

**Figure 4:**
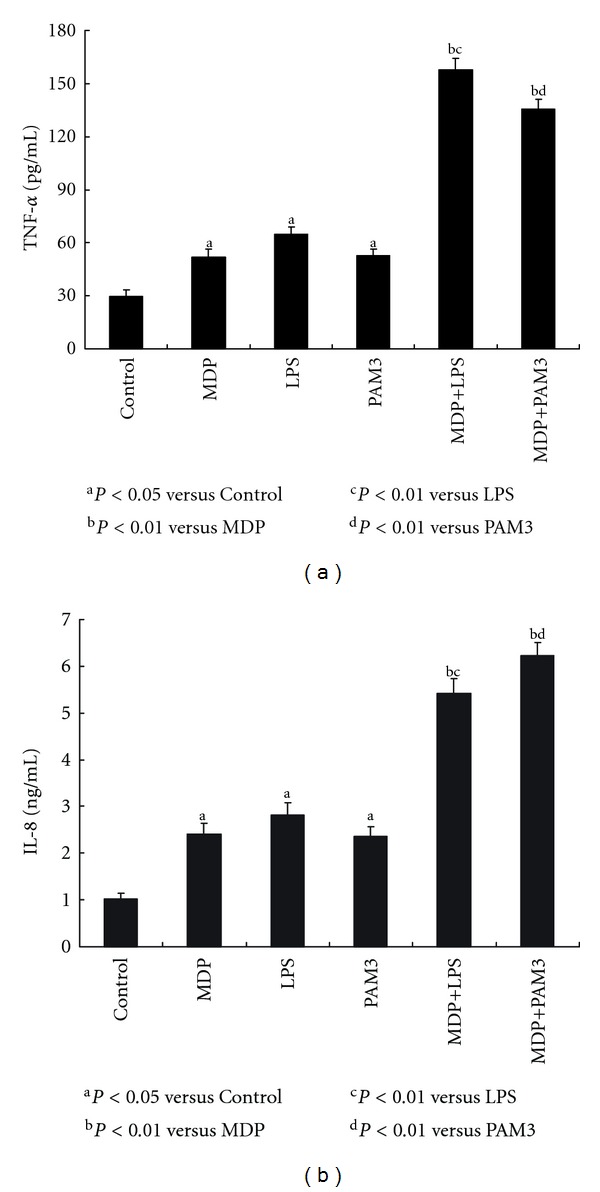
Inflammatory factors production by VSMC in response to MDP alone or in synergy with LPS and PAM3. VSMC have been stimulated by MDP, LPS, and PAM3 alone or in combination for 24 h. IL-8 and TNF-*α* concentration in cell culture supernatant were measured by ELISA. (a) concentration of TNF-*α* in supernatant. MDP, LPS, or PAM3 alone can stimulate TNF-*α* secretion (MDP: 51.9 ± 4.73 pg/mL; LPS: 64.7 ± 4.21 pg/mL; PAM3: 52.7 ± 3.58 pg/mL; control: 29.4 ± 3.73 pg/mL; ^a^ *P* < 0.05 versus control, *n* = 3). The combination of MDP with LPS or PAM3 had stronger stimulation effect. (MDP + LPS: 157.6 ± 6.68; MDP + PAM3: 135.6 ± 5.34; ^b^ *P* < 0.01 versus MDP; ^c^ *P* < 0.01 versus LPS; ^d^ *P* < 0.01 versus PAM3; *n* = 3). (b) concentration of IL-8 in supernatant. MDP, LPS, PAM3 alone can stimulate IL-8 secretion (MDP: 2.41 ± 0.22 ng/mL; LPS: 2.82 ± 0.26 ng/mL; PAM3: 2.36 ± 0.21 ng/mL; control: 1.02 ± 0.13 ng/mL; ^a^ *P* < 0.05 versus control, *n* = 3). The combination of MDP with LPS or PAM3 had stronger stimulation effect. (MDP + LPS: 5.41 ± 0.32 ng/mL; MDP + PAM3: 6.23 ± 0.28 ng/mL; ^b^ *P* < 0.01 versus MDP; ^c^ *P* < 0.01 versus LPS; ^d^ *P* < 0.01 versus PAM3; *n* = 3).

We tested the capacity of MDP, LPS, or PAM3 treatment alone or in combination to VSMC for production of inflammatory factors. RT-PCR analysis was done to determine FGF-2 mRNA expression in VSMC. The concentration of IL-8 and TNF-*α* in cell culture supernatant was measured by ELISA. The results showed although MDP, LPS, or PAM3 stimulation alone can cause increased FGF-2 mRNA expression, the combination of MDP with LPS or PAM3 did not show synergistic effect on FGF-2 mRNA expression (data not shown). However, the combination treatment had coactivating effect on concentration of IL-8 and TNF-*α* than each treatment alone, indicating MDP stimulates secretion of inflammatory factors in synergy with LPS and PAM3 (see Figures 4(a) and 4(b)).

## 4. Discussion 

Proliferation, hypertrophy, migration to the intima and synthesis of extracellular matrix of vascular smooth muscle cells after activation are characteristic of hypertension, atherosclerosis, restenosis, and other cardiovascular diseases, which are also the key pathological features of vascular remodeling [4]. More and more evidence indicate the engagement and the important role of infection and immune response in this process. In this study, we found that MDP can stimulate human coronary artery VSMC to up regulate expression of NOD2 mRNA. In addition, MDP increased FGF-2 mRNA expression in VSMC, IL-8, and TNF-*α* secretion in cell culture supernatant, and proliferation ability of VSMC. MDP is the degradation product of peptidoglycan (PGN) in most G^+^ and some G^−^ bacterial and is an NOD2-specific agonist, suggesting that some bacterial products can enter the VSMC, upregulate and activate intracellular NOD2, promote cell proliferation, and induce VSMC to produce inflammatory cytokines. NOD2 is the key member of a recently discovered NOD family of PRRs. Its activation can initiate the activation of the innate immune defense response of the host cells to invading pathogens and represent an intracellular defense and monitoring pathway [5]. Our findings confirmed the existence of NOD2-mediated innate immune signaling pathways in VSMC, which can be activated by pathogen to regulate the proliferation of VSMC and stimulate VSMC to secret inflammatory cytokines.

Our study also found that the capacity of MDP in combination with TLR4 agonist LPS or TLR2 agonist PAM3 to stimulate proliferation of VSMC and secretion of IL-8 and TNF-*α* was more potent than that of each reagent alone. Although synergistic FGF-2 production in VSMC was not observed with these agonists combination, these findings strongly imply a cooperative effect between NOD and TLR signaling. As the two important component of the innate immune system against pathogens, the relationship between TLRs and NODs remains controversial. Watanabe et al. [6] found that NOD2 negatively regulates TLR2-mediated (T helper type 1 response), while Uehara et al. [7] and Fritz et al. [8] reported that NOD1, 2 can coactivate monocytes with TLR2, 4. This study showed that TLR and NOD signaling can be costimulate VSMC to proliferate and produce some inflammatory cytokines.

The vital function of TLR2, 4-mediated signaling pathway in VSMC activation and atherosclerosis formation has been confirmed [9, 10], whereas the role of NODs in these field remain to be elucidated. This study showed MDP can induce FGF-2 production in VSMC and increased secretion of IL-8 and TNF-*α* in cell culture supernatant. FGF-2 is produced by VSMC, participates in proliferation, hypertrophy, migration to the intima and synthesis of extracellular matrix of VSMC after activation as a powerful mitogen, and plays crucial role in atherosclerosis [11]. TNF-*α* is a multipotential mediator in inflammatory reaction and contributes to development of atherosclerotic lesion and plaque stability through regulating proliferation and apoptosis of VSMC [12]. IL-8 is a strong chemotactic factor for neutrophils, monocytes, and T cells and an effective predictor of cardiovascular events after PCI [13]. Our results indicate NOD2-mediated innate immune signaling pathway probably get involved in atherosclerosis formation by stimulating VSMC to produce some inflammatory cytokines.

 Currently, PRR-mediated chronic inflammation is a determinant for the development and progression of chronic diseases including atherosclerosis formation, vascular remodeling, and cancer, however, the exact mechanism is still unclear [14]. In this study, we found that intracellular PRR NOD2-mediated innate immune signaling pathways can promote the proliferation of VSMC and induce VSMC to secret inflammatory cytokines, and function in synergy with TLR-mediated innate immune signaling pathway. Although the specific mechanism remains to be explored, these findings further confirm the engagement of infection and immune response in vascular remodeling and the formation of atherosclerosis understanding and shed light on the new strategies for prevention and control of coronary heart disease.
